# Stereotactic Dorsolateral Irradiation of Spinal Nerve Roots: A Novel Technique for the Treatment of Spasticity and Pain

**DOI:** 10.7759/cureus.8323

**Published:** 2020-05-27

**Authors:** Pantaleo Romanelli, Giancarlo Beltramo

**Affiliations:** 1 Neurosurgery, Cyberknife Center, Centro Diagnostico Italiano, Milano, ITA; 2 Radiation Oncology, Cyberknife Center, Centro Diagnostico Italiano, Milano, ITA

**Keywords:** selective dorsal rhizotomy, spasticity, pain, image-guided, robotic, stereotactic radiosurgery, frameless, radiation, spine, nerve root

## Abstract

Selective dorsal rhizotomy is an established surgical treatment to improve the neurological and functional status of children with spastic cerebral palsy and adults with spasticity and pain caused by a variety of brain and spinal injuries. This procedure requires a dorsolumbar laminectomy to expose the appropriate dorsal rootlets, which are sectioned according to the presence of sustained electromiographic discharges.

Image-guided robotic radiosurgery targeting the intracisternal sensory root of the trigeminal nerve has been described as a safe and effective non-invasive treatment for trigeminal neuralgia, a paroxystic pain disorder which often responds poorly to medical therapy. Image-guided radiosurgery requires no frame placement and can treat brain or spinal targets with submillimetric precision. This technique can be used to treat cervical or lumbar dorsal roots.

A 44-year-old patient with von Hippel-Lindau disease developed severe spastic tetraparesis following multiple brain and spinal procedures. Spasticity and related pain mostly affected the right leg, with sustained electromiographic discharges originating from the right L4 nerve root. Response to medical therapy with baclofen and cannabinoids was poor. Due to geographical and logistical issues, the patient declined the placement of an intrathecal baclofen pump.

Considering the poor general condition of the patient and his decision to avoid invasive procedures, a novel treatment option was offered to provide relief from spasticity and pain: stereotactic image-guided irradiation delivered to the sensory root. The patient underwent a right intraforaminal dorsolateral L4 root stereotactic irradiation with a delivered dose of 45 Gy prescribed to the 82% isodose.

The treatment was well tolerated, without side effects. Resolution of spasticity and related pain in the right leg was found six months after the procedure. A marked reduction of spasticity and pain was also evident in the contralateral leg. These improvements have been stable over the last 18 months.

So far, two additional patients underwent stereotactic dorsolateral spinal root irradiation (one delivered to a cervical, the other to a lumbar), with similar positive outcomes. These preliminary results suggest that spinal root stereotactic image-guided irradiation, a novel treatment option in the neurosurgical armamentarium, is a safe and effective procedure and deserves further investigation.

## Introduction

This paper introduces a novel non-invasive treatment for spasticity: stereotactic dorsolateral irradiation of spinal nerve roots. This new treatment was developed to offer the radiosurgical equivalent of selective dorsal rhizotomy (SDR). SDR is an established procedure to treat spasticity in children and adults [1-2]. Spasticity originates from the failure of upper motor neurons to control the discharge of lower motor neurons. The increased firing by the lower motor neurons following the loss of supraspinal control conveyed by the corticospinal, reticulospinal, and vestibulospinal tracts leads to the increased muscle tone that characterizes spasticity. SDR counteracts spasticity by removing the afferent inputs of the reflex arc, brought to the spinal motor neurons by the sensory fibers (Ia) located in the dorsal spinal roots. SDR is a relatively invasive procedure requiring the opening of the dural sac with exposure and section of the involved rootlets. The selection of the rootlets to be sacrificed is guided by intraoperative electrophysiological mapping. Pre-operative botulinum injections are also useful to identify the dorsal roots involved in the genesis of spasticity and to predict the clinical efficacy of the procedure. An alternative to SDR, especially in adult patients, is the placement of an intrathecal baclofen pump. The main shortcoming of this procedure is the need to periodically refill the reservoir: missing baclofen refill appointments can lead to severe withdrawal syndrome culminating with seizures and death.

Stereotactic irradiation is a well-established procedure for the treatment of trigeminal neuralgia (TN). The target of stereotactic irradiation is the sensory root of the trigeminal nerve. CyberKnife® (Accuray Incorporated, Sunnyvale, California) frameless image-guided robotic radiosurgery is currently the less invasive non-medical treatment for TN, providing robust and durable pain control and absence of major neurological complications [3-4]. CyberKnife® radiosurgery can be used safely in any body region, allowing the precise and accurate treatment of targets unreachable by other radiosurgical platforms. Cervical or lumbar sensory roots can be treated safely by CyberKnife® radiosurgery.

The aim of this case report is to describe the use of CyberKnife® stereotactic image-guided irradiation of spinal nerve roots as a novel non-invasive treatment option for spasticity.

## Case presentation

A 44-year-old man with severe spasticity and pain affecting the right leg underwent stereotactic image-guided irradiation of the dorsolateral right L4 root. The anamnesis of the patient was remarkable for von Hippel-Lindau disease with recurrent brain and spinal haemangioblastomas. He underwent several surgical and radiosurgical procedures starting when he was 17 years old, including the resection of intracranial (bulbar) and spinal (thoracic) lesions, and the radiosurgical treatment of recurring lesions located in the posterior fossa (fourth ventricle), the cervical spine at the C3-C4 level, the perimesencephalic region (right cerebral peduncle), the cervical spine again at the C6-C7 level, and the thoracic spine at the T3 level.

The patient emerged from the first surgical procedure with a right-sided hemiparesis. Over time, he developed tetraparesis with severe spasticity and pain, mostly affecting the right leg, and became wheelchair-bound. Medical therapy including baclofen and cannabinoids failed to achieve satisfactory results, with spasticity-related pain in the right leg being very intense. The implant of an intrathecal baclofen pump was suggested but the patient refused to undergo further invasive procedures. In any case, no local provider for the baclofen pump refill was found. Based on electromyography (EMG) findings showing a sustained increase of the right L4 root discharges, CyberKnife® steretotactic image-guided robotic irradiation of the dorsolateral intraforaminal right L4 root was performed. 

**Figure 1:**
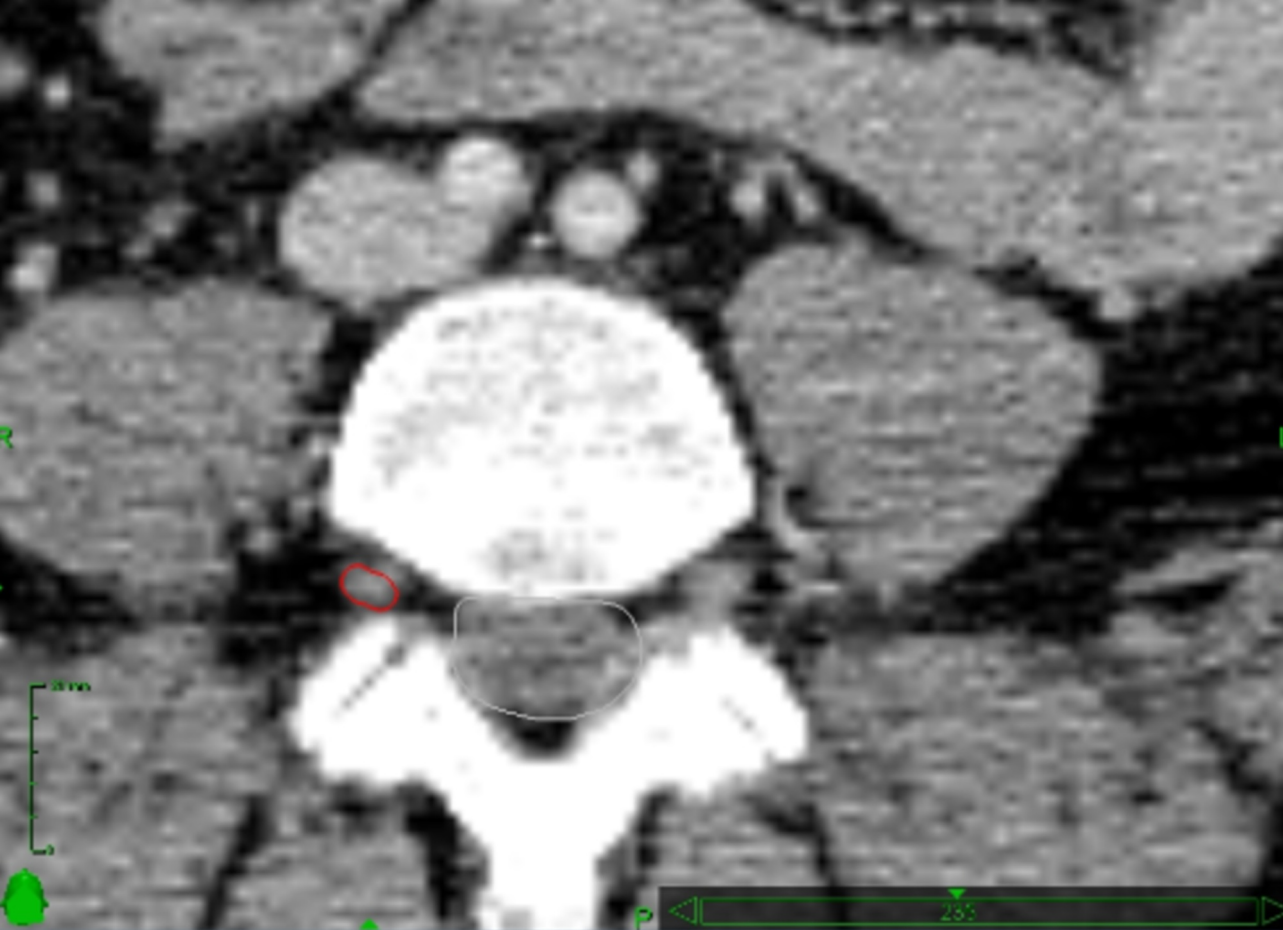
Target identification in the CT scan used for treatment planning The dorsolateral segment of the right L4 nerve root is contained within the red line. The target is located inside the L4 foramen.

Figure 1 shows the target identified on CT scan.

**Figure 2:**
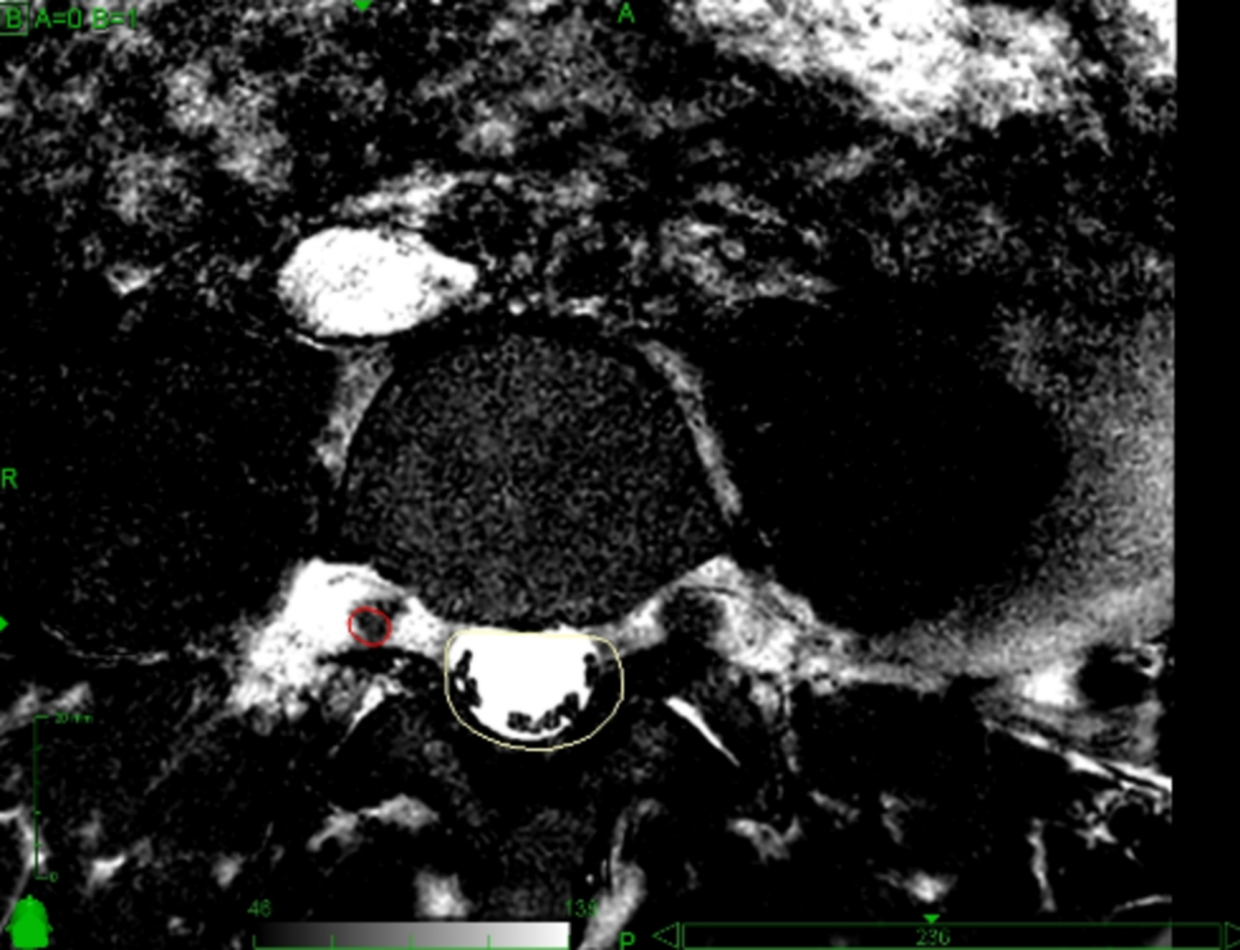
MR target identification Fast imaging employing steady-state acquisition (FIESTA) sequence was used to identify the target. The dorsolateral part of the right L4 root is visible inside the red line.

Figure 2 shows the MR identification of the right dorsolateral L4 root.

**Figure 3:**
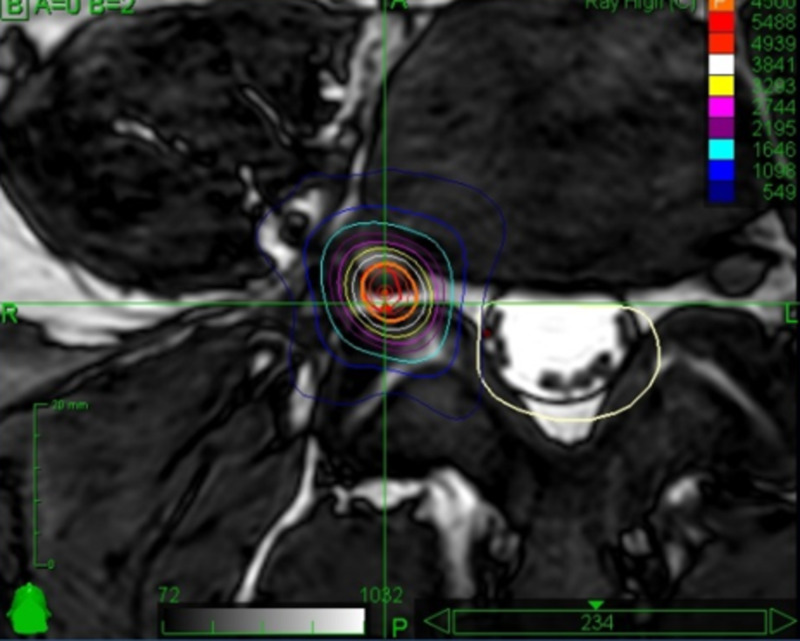
Axial screenshot of the treatment planning delivering stereotactic irradiation to the right L4 intraforaminal root

Figure 3 shows the axial screenshot of the treatment planning.

A 5 mm dorsolateral segment of the nerve root received 45 Gy prescribed to the 82% isodose. The mean delivered dose was 50.24 Gy and the maximum dose was 54.87 Gy. The target volume was 82 mm³. The procedure was well tolerated, without side effects or complications. Spasticity and pain decreased slowly over the following weeks. The pain disappeared completely within three months, going down from 7 to 0 on the visual analogue scale (VAS). Spasticity in the right leg was markedly reduced after six months, going down from a score of 5 to a score of 1 on the Modified Ashworth Scale (MAS). The MAS score in the contralateral leg improved as well, going down from a score of 4 to a score of 2. After 18 months, both pain and spasticity in the right leg remain absent. No hypoesthesia was detected. Upon independent assessment, the patient and his family expressed great appreciation for the results obtained through the non-invasive procedure described above. 

## Discussion

Spasticity is a common sensorimotor disorder following either brain or spinal cord injury, compromising the corticospinal, reticulospinal, and vestibulospinal pathways. Loss of the inhibitory control exercised by the supraspinal motor neurons upon the spinal motor neurons gives way to uncontrolled stimulation of the latter by the reflex arc with consequent enhanced, sustained firing leading to increased muscle tone (hypertonus), increased spinal stretch reflex (hyperreflexia), clonus, and painful muscle spasms in response to stretch and noxious cutaneous stimulation.

Traumatic brain and spinal cord injuries are the most common cause of spasticity in the adult population [2]. Close to 100 million people worldwide suffer the sequelae of severe trauma to the brain or spinal cord, with spasticity and related pain being a common long-term complication in survivors [2].

Oral antispasmodic agents such as baclofen, botulinum injections, physiotherapy, and occupational therapy are the most common measures taken to improve spasticity but overall results are often unsatisfactory. Aside from orthopedic intervention, the neurosurgical armamentarium offers the possibility to implant an intrathecal baclofen pump or to proceed with SDR.

The most common indication for SDR is the treatment of spastic diplegia induced by cerebral palsy in children, with class I evidence supporting its efficacy [5]. SDR has also been sporadically used in adults to treat spasticity and related pain caused by trauma, multiple sclerosis, stroke, congenital brain malformations, and hereditary spastic paraparesis [1]. Overall, the reported results are favorable and complications are rare [1-2, 5-6].

The placement of an intrathecal baclofen pump is an alternative to SDR but hardware-related complications are relatively common. Lack of refilling of the baclofen reservoir can lead to an abrupt and potentially life-threatening withdrawal syndrome. Also, the cost and maintenance of the device can be an issue in several countries.

As shown by this case-report, radiosurgical irradiation of the nerve roots involved in the generation of spasticity is an interesting non-invasive alternative to SDR or to the placement of a baclofen pump. The procedure described here was well-tolerated and achieved excellent control of spasticity and pain. It should be noted that the dose delivered to this patient was not strictly an ablative dose. The clinical benefit is likely to be mediated by the non-necrotic effects of radiation. Radiomodulation, an elusive and poorly studied phenomenon, is possibly related to the symptomatic relief seen after radiosurgery of cranial or spinal nerve roots, as clearly shown by the fact that facial hypoesthesia affects only a small share of TN patients experiencing symptomatic relief following radiosurgical treatment [3-4].

If the clinical benefit produced by the irradiation of cranial or spinal nerve roots is indeed generated by a non-ablative process, we can speculate that image-guided frameless radiosurgery, a precise, non-invasive, and highly cost-effective procedure, should be investigated further as a tool to treat functional disorders (such as pain, epilepsy, spasticity, etc.) affecting the central and peripheral nervous systems.

## Conclusions

Stereotactic irradiation of the right L4 nerve root was performed on a 44-year-old patient with severe spasticity affecting the right leg. Treatment was focused on this specific root on the basis of the indications provided by EMG. The procedure was well tolerated and no complications were observed. Spasticity and pain decreased steadily over time. After six months, muscle tone in the right leg was close to normal and no pain was present. These findings are stable 18 months after the procedure. These encouraging preliminary results pave the way for a larger prospective study on the safety and efficacy of EMG-guided spinal roots stereotactic irradiation for the treatment of spasticity in adults.

## References

[1] Gump WC, Mutchnick IS, Moriarty TM (2013). Selective dorsal rhizotomy for spasticity not associated with cerebral palsy: reconsideration of surgical inclusion criteria. Neurosurg Focus.

[2] Agrawal M, Samala R, Doddamani R, Agrawal D, Chandra SP (2020). The role of selective dorsal rhizotomy in the management of post-traumatic spasticity: systematic review (IN PRESS). Neurosurg Rev.

[3] Romanelli P, Conti A, Bianchi L, Bergantin A, Martinotti A, Beltramo G (2018). Image-guided robotic radiosurgery for trigeminal neuralgia. Neurosurgery.

[4] Romanelli P, Conti A, Redaelli I, Martinotti AS, Bergantin A, Bianchi LC, Beltramo G (2019). Cyberknife radiosurgery for trigeminal neuralgia. Cureus.

[5] Park TS, Dobbs MB, Cho J (2018). Evidence supporting selective dorsal rhizotomy for the treatment of spastic cerebral palsy. Cureus.

[6] Enslin JMN, Langerak NG, Fieggen AG (2019). The evolution of selective dorsal rhizotomy for the treatment of spasticity. Neurotherapeutics.

